# Screen for MicroRNA and Drug Interactions in Breast Cancer Cell Lines Points to miR-126 as a Modulator of CDK4/6 and PIK3CA Inhibitors

**DOI:** 10.3389/fgene.2018.00174

**Published:** 2018-05-18

**Authors:** Federica Baldassari, Carlotta Zerbinati, Marco Galasso, Fabio Corrà, Linda Minotti, Chiara Agnoletto, Maurizio Previati, Carlo M. Croce, Stefano Volinia

**Affiliations:** ^1^Laboratory for Technologies of Advanced Therapies (LTTA), Department of Morphology, Surgery and Experimental Medicine, University of Ferrara, Ferrara, Italy; ^2^Department of Cancer Biology and Genetics, The Ohio State University Wexner Medical Center, Columbus, OH, United States

**Keywords:** cell cycle, LEE011 (Ribociclib), BYL719 (Alpelisib), miRNA, non-coding RNA

## Abstract

**Background:** Breast cancer (BC) represents the most common cancer in women worldwide. Due to its heterogeneous nature, breast cancer management might benefit from differential treatments toward personalized medicine. Additionally, drug resistance is a common phenomenon. We systematically investigated the effect of 14 different drugs administered on BC cell lines in combination with microRNAs (miRNA, miR).

**Methods:** Thirty-eight miRNAs, all associated with BC by clinical and molecular parameters including progression, prognosis and subtypes, were tested for their effects on the viability of 12 different BC cell lines. Four miRNAs with the strongest impact on viability were further assayed in combination with 14 BC drugs. Mann–Whitney *U*-test with Bonferroni correction was used for statistical analysis.

**Results:** In a miRNA only pre-screen we observed effects on BC cell lines' viability for 34 out of 38 candidate miRNAs. We then identified 14 miRNA/drug combinations for which the combination IC_50_ was lower than that of both miRNA and drug as single agents. miR-181a, paired with GSK1070916, Doxorubicin, XL765 and AMG511, was the only miRNA active on the triple negative (TNBC) MDA-MB-468 cell line. miR-126 was the only miRNA (in combination with CDK4/6 or PIK3CA inhibitors) with significant effects on cell lines from different subtypes: MCF7 (Luminal) and MDA-MB-453 (HER2^+^). Because of its activity on different BC subtypes, we investigated the genome wide effects of miR-126 using transcriptomics and confirmed that expression of miR-126 in BC cell lines affected cell cycle and mitosis.

**Conclusion:** Our results show that a combination treatment with miRNAs, in particular miR-181a, miR-326, miR-9 and miR-126, enhance the activity of specific BC drugs *in vitro*, even on the most aggressive BC subtypes, HER2+ and TNBC. Finally, as expected from its drug interactions, based on a whole transcriptome study we could confirm a role for miR-126 in cell cycle regulation.

## Introduction

Breast cancer (BC) is the most common cancer in women worldwide representing about 25% of all cancers, with nearly 1.7 million new cases diagnosed in 2012 (second most common cancer overall). This corresponds to about 12% of all new cancer cases and 25% of all cancers in women as reported by the World Cancer Research Fund International website^1^ Its incidence has been predicted to rise further do to behavioral and environmental changes that greatly affect the main risk factors of BC (Arnold et al., 2015).

Breast cancer is a complex and heterogeneous disease, characterized by variant genetic alterations and distinct morphologic and molecular features. The assessment of estrogen receptor alpha (ER), progesterone receptor (PR), and human epidermal growth factor receptor-2 (HER2) status, in association with gene expression profiling revealed a subtype classification of BC. The classification includes luminal A (ER+/PR+, HER2–), luminal B (ER+/PR+, HER2+), HER2^+^-enriched (HER2 positive), and Basal-like or Triple-Negative (ER–, PR–, HER2–) (Perou et al., 2000; Sotiriou et al., 2003; Prat et al., 2015). Each subtype has different prognosis, needs appropriate treatment and displays specific response (Sørlie et al., 2001).

Nowadays there is an acceleration toward implementation of individualized treatment and thus an ever larger need for the widest possible range of therapeutic tools. Notwithstanding more sensitive diagnostics and advances in early detection, drug resistance is still one of the major problems that remain to be solved. Too often, in fact, patients go on to develop aggressive malignancies, which exhibit resistance to one or more drugs. The underlying mechanisms of acquired resistance to chemotherapeutic agents being still poorly understood.

MicroRNAs, among the most investigated actors in the non-coding RNA (ncRNA) panorama, can potentially be used to increase the response of cancer to therapeutic interventions (Chen et al., 2017). The involvement of non-coding RNAs has represented a crucial discovery in molecular mechanism of cancer and indicated novel potential biomarkers for diagnosis and prognosis of BC (Baker, 2010; Bertoli et al., 2015). microRNA (miRNA, miR) is a class of short conserved non-coding RNAs, ~22 nucleotides in length, present in eukaryotic cells (Lagos-Quintana et al., 2001; Bartel, 2009) and exerting important roles in cancer (Croce, 2008). In 2005, Iorio and co-workers identified differentially expressed miRNAs in clinical subgroups of BC (Iorio et al., 2005). Additional studies reported clinically relevant roles for miRNAs in BC (Foekens et al., 2008; Rothé et al., 2011), demonstrating that expression of miRNAs can provide independent information for clinical and prognostic usage in patient management (Galasso et al., 2012).

The effects of miRNAs on drug resistance in BC have already commenced to be addressed (Kutanzi et al., 2011; Wang J et al., 2015) but still to little extent and the relative miRNAs' wherewithal remains largely to be determined.

Building on these premises, we decided to systematically investigate the effects of a rationally selected subset of miRNA in several BC cell lines, by themselves or in association with 14 different drugs. Some reports showed a clear role for miRNAs in modulation of drug resistance (Ayers and Vandesompele, 2017) but our work introduces in this scenario additional miRNAs and drugs, also in relation to the different BC subtypes.

## Methods

### Cell culture and miRNA transfection

The human breast cancer cell lines were purchased from the American Type Culture Collection (ATCC). MDA-MB 468, MCF7, ZR75.1, MDA-MB-361, and MDA-MB-231 were cultured in Dulbecco's modified Eagle's medium (DMEM, Sigma-Aldrich), while SKBr3 in McCoy's (Lonza). All media were supplemented with 10% fetal bovine serum (FBS), 1% penicillin and streptomycin antibiotics (PS) and 1% L-glutamine. T47D, MDA-MB-453, BT474 and HBL100 were maintained in RPMI-1640 medium with L-glutamine (Sigma-Aldrich) supplemented with 10% FBS and 1% PS. Immortalized MCF-10A and 184A1 cells were cultured in mammary epithelial cell growth medium F12 (Gibco) supplemented by 10% FBS, 1% PS, 10 μg/ml bovine insulin, 100 ng/ml cholera toxin, 20 ng/ml recombinant human epidermal growth factor, 0.5 μg/ml hydrocortisone (SIGMA, St. Louis, MO). All cell lines were grown in a 37°C humidified incubator with 5% CO_2_ and tested negative for mycoplasma.

The cells were transiently transfected with miRNA mimics (Ambion, USA) using siPORT™ NeoFX™ Transfection Agent (Invitrogen, USA) as recommended in the instruction protocol. Briefly, we operated a dilution of miRNA and siPORT™ NeoFX™ in separate tubes containing medium without FBS. Upon mixing, the transfection complex was incubated at RT (room temperature) for 10 min, and then transferred to a 96-well plate containing cells in appropriate medium with 0.1% FBS. Final miRNA concentration for transfection was 100 nM.

### Cell viability and miRNA assays

The MTS assay was performed to assess cell viability after miRNA transfection in 0.1% FBS. MTS (3-(4,5-dimethylthiazol-2-yl)-5-(3-carboxymethoxyphenyl)-2-(4-sulfophenyl)-2H-tetrazolium) CellTiter 96 Aqueous Reagent Powder (PROMEGA, Madison, USA) and PMS (phenazine methosulfate) (SIGMA), were diluted in phosphate buffered saline (PBS) in a 20:1 ratio and used to assess cell proliferation according to the manufacturer's protocol. Briefly, 6 × 10^3^ cells, for each BC cell lines, or 12 × 10^3^ cells, for breast epithelium cell lines (MCF10A and 184A1) were seeded into 96-well flat bottom microplates in a final volume of 100 μl, with 0.1% FBS, per well. The MTS/PMS mix was added to each well and the microplates further incubated for 1–4 h at 37°C and 5% CO_2_. The absorbance (OD) of the samples was read with a SUNRISE^TM^ ELISA plate reader operating at 492 nm (Tecan, Mannedorf, CH). All experiments were repeated at least three times in duplicates.

AlamarBlue cell viability assays were performed as recommended in the instruction protocol. Briefly, for all BC cell lines, 6 × 10^3^ cells were seeded into 96 wells plates. The reagent was added in 1:10 proportions to the cells in culture. After 4–6 h of incubation at 37°C, absorbance was monitored at 570 and 620 nm wavelengths. Again, as for MTS assays, all experiments were repeated at least three times in duplicates.

### miRNA/drug interactions

Drug sensitivity was specifically investigated to determine the inhibition dose for each cell line. Cell viability assays were performed in triplicates with alamarBlue. MDA-MB-468, T47D, MCF7, and MDA-MB-453 were plated at 6 × 10^3^ cells/well in 96-well plates and treated with increasing concentration of drug in a range starting from 1.28 nM up to 100 μM, or from 0.64 nM up to 1 μM for Docetaxel, in 10% FBS medium, for 72 h. We determined the inhibition curves using Graph Prism. To evaluate possible synergisms or interactions between miRNAs and drugs, cells were also transfected using siPORT™ NeoFX™ with miRNAs final concentration of 50 nM in 10% FBS medium. After 16 h, the medium was changed and the drug added. Passed 72 h, the alamarBlue cell viability assay was performed. Experiments were performed at least in duplicate for three times.

### Statistics and data mining

For the analysis of MTS experiments we calculated the ratio between the mean (quadruplicate samples) of each miRNA and the global median from all transfections. We then calculated, for each miRNA, the absolute deviation from the median, i.e., MAD. We deemed significant the effect of a miRNA for which the median MTS-value was larger (or smaller) than the global median +/- 2 times its MAD. The Mann–Whitney *U*-test was used for the alamarBlue assay (cell lines treated with miRNA and/or drug in combination). The Bonferroni correction was applied, dividing the critical *P*-value (α = 0.05) by the number of comparisons for each experiment. Normalized miRNA profiles (*n* = 796) for the METABRIC study (Dvinge et al., 2013) were obtained from the European Genome-phenome Archive (EGA) (accession number EGAD00010000438). The TCGA miRNA profiles for primary breast cancers were obtained from TCGA data portal (*n* = 918).

### Human transcriptome and cellular pathway analysis

Breast cancer data for TCGA mRNA were obtained from Firehose web site (https://gdac.broadinstitute.org/). Lentiviral assays for miR-126 were obtained from GEO (GSE40458). Filtering was enabled to filter out genes that had <20% of expression data with at least a 1.5-fold change in either direction from gene's median value. Correlation between miRNAs and mRNAs was performed using Spearman correlation. The genes for the regulated mRNAs were studied for functional enrichment on PantherDB (http://pantherdb.org/) and Gene Set Enrichment Analysis (Wang et al., 2013a).

## Results

### miRNA selection

The key criteria for the inclusion of a miRNA in our study were based on microRNA profiles in BC cohorts: (i) differentially expressed miRNAs in solid tumors vs. normal breast samples (TCGA), and (ii) miRNAs related to the transition from Ductal Carcinoma *In Situ* (DCIS) to Invasive Ductal Carcinoma (IDC) (Supplementary Table 1A; Volinia et al., 2010, 2012, 2014). Additional miRNAs included in this study and related to prognosis of BC were obtained using METABRIC and TCGA clinical data (Supplementary Table 1B; Martello et al., 2010; Tang et al., 2012; Volinia and Croce, 2013; Wang et al., 2013b; Li et al., 2014).

### miRNA effect on cell proliferation

**Figure 1:**
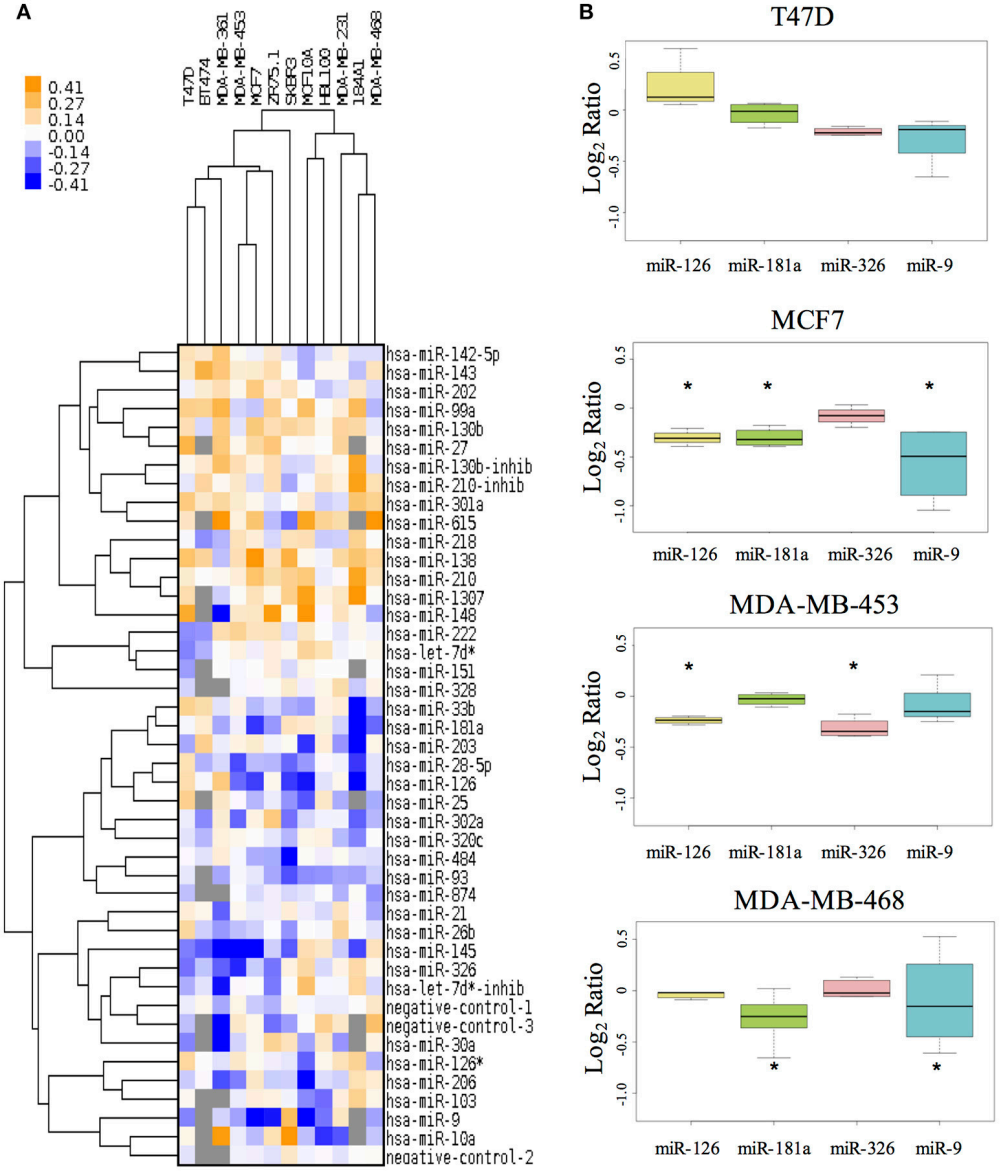
The effect of miRNAs on cell proliferation of breast cancer cell lines. **(A)** The MTS assay reveals the effects of miRNAs on cell proliferation in 10 different BC cell lines and in 2 non-tumorigenic breast cell lines (MCF10A and 184A1). The tree with the cluster analysis shows miRNA proliferative effects (in orange) and anti-proliferative effects (in blue). **(B)** The boxplot reports in detail the results for four miRs and four cell lines. *Indicates miRNA's effect higher/lower than global median plus/minus 2 MADs.

Prior to cell assays we tested the transfection efficiency of MDA-MB-453, MDA-MB-468, MCF7, and T47D cell lines using siPORT and a plasmid containing the green fluorescence protein EGFP. After 48 and 72 h the mean efficiency of transfection for all cell lines explored was acceptable and comparable, 70 ± 10% (data not shown) and prompted us to carry on with the experiment. We then investigated the miRNA effects on the proliferation of 10 breast cancer cell lines and of 2 cell lines derived from normal breast epithelium. This analysis was performed to gain experimental evidences on the miRNA functional involvement in cancer mechanisms, where often the growth signals are constitutively activated by a host of mutations (e.g., PIK3CA mutation, HER2 amplification, CDKN2A deletion). We thus assayed cell proliferation upon miRNA transfection in condition of serum deprivation (0.1% FBS). Figure 1A shows the results for each miRNA in each cell line: orange color indicates proliferative effect, and blue anti-proliferative, after 48h from transfection. The MTS tests indicated that 24 miRNAs had significant effects in at least two cell lines. Among them miR-26b, miR-99a, miR-130b, miR-138, miR-143, miR-210, miR-1307, miR-615, miR-484, miR-27, miR-301a, and miR-148b increased cell viability. Conversely, miR-145, miR-28-5p, miR-126, miR-181a, miR-203, miR-206, miR-326, miR-103, miR-93, miR-30a, miR-9, and miR-874 decreased cell viability.

Thus, we selected 4 of the miRNAs with higher anti-growth effect as candidate enhancer of anticancer drugs (miR-9, miR-126, miR-181a, and miR-326). Figure 1B shows the effect of these 4 miRNAs on cell lines representing different BC subtypes: T47D and MCF7 for Luminal, MDA-MB-453 as HER2^+^ and MDA-MB-468 as Triple-Negative.

### Cell line specific drug sensitivity

We used 14 cancer drugs with different targets of action to be combined with the 4 active miRNAs in subsequent studies for miRNA-drug interactions. Most of these drugs have been assessed in clinical trials^2^^,^^3^, as summarized in Table 1.

**Table 1:** Table shows al drugs used, their mechanisms of action and their involvement in clinical trials against breast cancer or other tumors^2^^,^^3^.

**Drug**	**Synonym**	**Target**	**Trials**	
			**BC**	**Others**
AZD5363		PKB/AKT isoforms inhibitor	✓	✓
AZD7762		ATP-competitive CHK1/2 inhibitor		✓
AZD8055		ATP-competitive mTORC1/C2 inhibitor		✓
BYL719	ALPELISIB	PI3K α-isoform (PIK3CA) specific inhibitor	✓	✓
ERLOTINIB	TARCEVA	Epidermal Growth Factor Receptor (EGFR) tyrosine kinases inhibitor	✓	✓
GEFITINIB	IRESSA	Epidermal Growth Factor Receptor (EGFR) tyrosine kinases inhibitor	✓	✓
GSK1070916		ATP competitive inhibitor of Aurora B and C kinases		✓
GSK1120212	TRAMETINIB	MEK 1/2 inhibitor	✓	✓
LEE011	RIBOCICLIB	Cyclin-dependent kinase 4 and 6 (CDK 4/6) inhibitor	✓	✓
SCH772984		ATP competitive inhibitor of ERK1 and ERK2		
DOXORUBICIN	ADRIAMICIN	DNA intercalant, TOPOiso II inhibitor	✓	✓
DOCETAXEL	TAXOTERE	Binds tubulin inducing cell-cycle arrest at the G2/M phase. Vascular endothelial growth factor (VEGF) inhibitor	✓	✓
AMG511		Selective pan-class I phosphatidylinositol-3 kinase (PI3K) inhibitor		
XL765	VOXATALISIB	Reversible ATP-competitive inhibitor of pan-Class I PI3K (α, β, γ, and δ) and mTORC1/mTORC2	✓	✓

*All drugs were purchased from Chemietek*.

**Figure 2:**
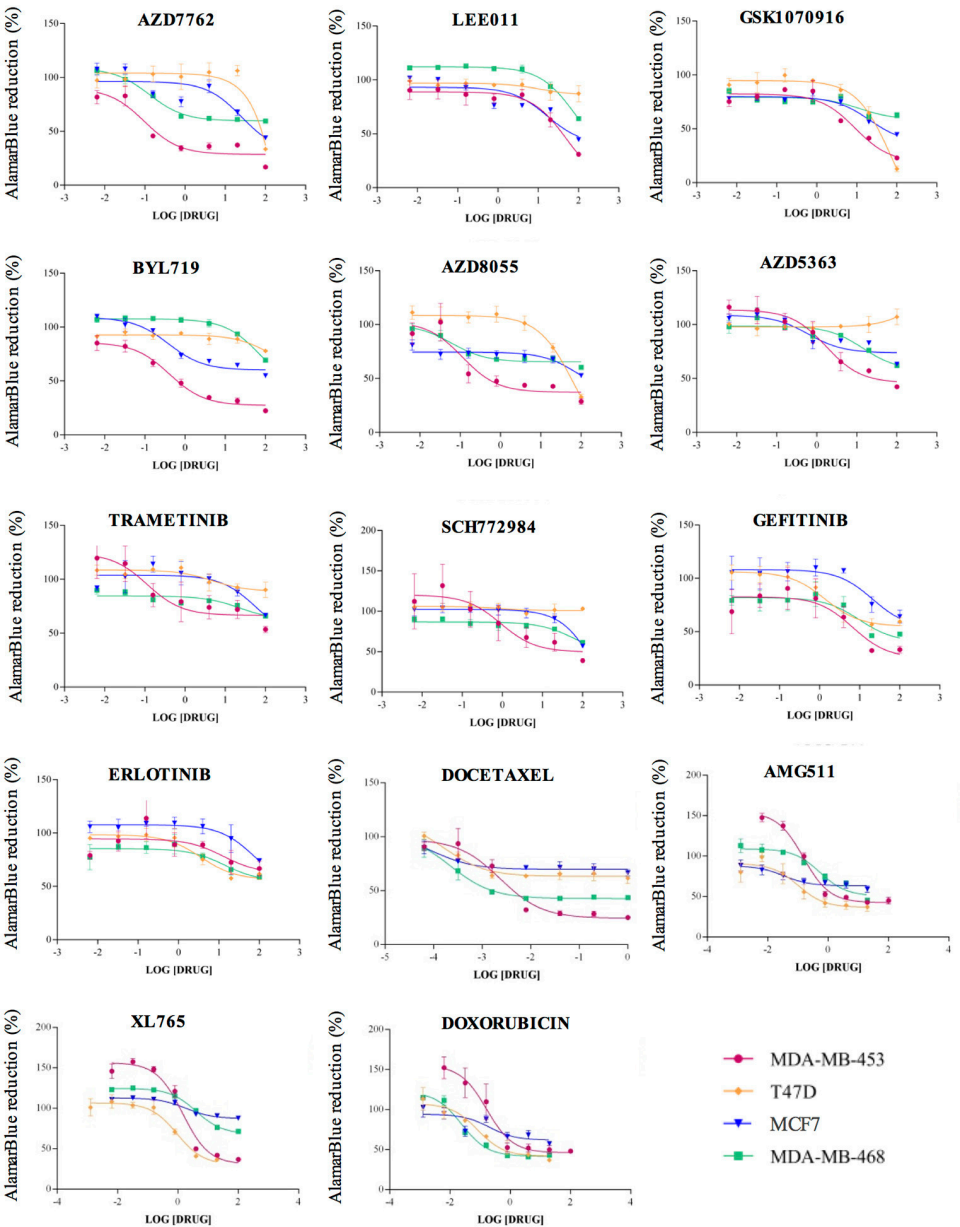
Drug sensitivity curves. For the drug sensitivity evaluation, each cell line (MDA-MB-453, T47D, MCF7 and MDA-MB-468) 0020 was treated with progressive concentrations of each drug in a range starting from 1.28 nM up to 100 μM, or from 0.064 nM to 1 μM for Docetaxel. We determined the inhibition curves, as assessed by alamarBlue, using Graph Prism. The drug concentration units indicated on the X axes are micromolar.

To determine cell line specific drug sensitivity (IC_50_), cell viability assays were performed using increasing drug concentrations. We treated all four BC cell lines with scalar concentration of each drug, in a range from 1.28 nM to 100 μM, or from 0.64 nM to 1 μM for Docetaxel; alamarBlue was used to evaluate cell growth inhibition (Figure 2). If IC_50_ was higher than 1 μM, 1 μM concentration of the drug was used in the miRNA/drug interaction assay. The experimental concentrations determined for the drug/miRNA assays are reported in Supplementary Table 2. Since some of these drugs target cancer genes mutated in BC, the most notable somatic mutations^4^ in the cell lines are reported in Supplementary Table 3.

### Drug and miRNA interaction assay

**Figure 3:**
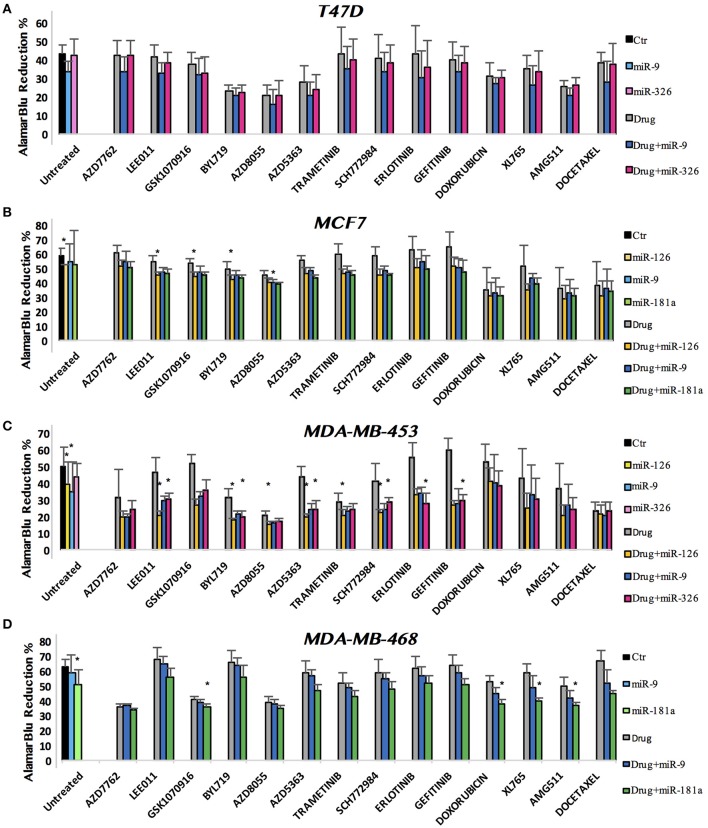
miRNA and drug combination affect the viability of breast cancer cell lines. alamarBlue reduction percentage as indicator of cell viability is shown. Results are reported as bar chart, indicating mean ± SD. For statistical analysis Mann–Whitney *U*-test has been used. We compered the *p*-value of three distinct analysis: single miR vs. Comb, single drug vs. Comb and untreated vs. Comb. When all *p*-values were minor than the Bonferroni corrected threshold (see section Methods), we defined the effect of combined miRNA/drug as a true interaction (*).

We finally combined miRNAs and drugs to investigate all possible interactions. A series of cell growth experiments was performed, with alamarBlue measured at 72 h after treatment with drug and miRNA in 10% FBS medium. Figure 3 shows the complete viability chart for the cell lines (in each row) and miRNA/drug combination. The untreated cells, or only miRNA-transfected controls, are on the left-hand side of the bar chart.

In most assays, the miRNA acted decreasing cell viability also in complete medium, as expected from our initial results with miRNA transfection in 0.1% FBS. For what concerns the miR-drug combinations, some pairs had remarkable effects. To identify them statistically, we operated 3-way comparisons: single miRNA vs. combined, single drug vs. combined and untreated vs. combined. The Bonferroni correction was applied, to yield stringent *p*-value thresholds for significance: α = 0.005 for MDA-MB-453 and MCF7 (each *n* = 10), and α = 0.007 for MDA-MB-468 and T47D (each *n* = 7). To generate robust results, we deemed the effect of combined miRNA/drug as a true interaction only if all *p*-values for the 3 comparisons were significant. For a stringent statistics the Bonferroni correction was applied also for the single miRNA effects: α = 0.017 for MDA-MB-453 and MCF7 (each *n* = 3), and α = 0.025 for MDA-MB-468 and T47D (each *n* = 2). Figure 3 shows a bar chart of all the treatments: we plotted bundles of bars corresponding to each drug. As explained above, in the first bundle on the left we reported the untreated control and the miRNA-only transfections. In the following bundles toward right-hand side, the first gray bar corresponds to the drug treatment, followed by the combination with each miR. In each row (cell line) the color dyes refer to a specific miR. The height of the bar reports the alamarBlu reduction that indicates cell count: lower alamarBlu reduction meaning viable cell count. T47D was the only cell line where we observed no significant interaction between miRNAs and cancer drugs (Figure 3A). In MCF7, miR-126 strengthened the effect of LEE011, GSK1070916, and BYL719, while miR-9 enhanced AZD8055 growth inhibition (Figure 3B). MDA-MB-453 showed interactions for six drugs, all involving miR-126: LEE011, BYL719, AZD8055, AZD5363, Trametinib, and SCH772984. miR-326 had interactions with the following drugs: LEE011, BYL719, AZD5363, SCH772984 (all 4 in common with miR-126), Erlotinib and Gefitinib (Figure 3C). In the MDA-MB-468 cell line, miR-181a interacted positively with GSK1070916, Doxorubicin, XL765, and AMG511 (Figure 3D).

Among the many combinations with positive effects against cell proliferation, the miR-126/LEE011 and miR-126/BYL719 pairs were significant (p < 0.005) in both cell lines we evaluated, namely MCF7 and MDA-MB-453. Therefore, we went on to treat with these combinations the remaining two cell lines, T47D and MDA-MB-468. But for these two additional cell lines miR-126, alone or in combination with LEE011 or BYL719, did not show significant effects (Supplementary Figure 1). The basal expression levels observed for miR-126 in the BC cell lines (Supplementary Figure 2) were very low (below 5 on a log2 scale and close to background, as compared for example to miR-221), as expected for breast cancer in comparison with normal breast, and did not prompted us to evaluate its knock-down. Our 3-way test was highly stringent, and there are a few drug/miRNA combinations that are still significant when the three tests were relaxed at *p* < 0.05. For example, miR-126 and XL765 were significantly more active in combination than alone both in MCF7 e MDA-MB-453.

### Gene ontology

To identify the cellular pathways and processes regulated by expression of miR-126 in BC, we correlated the levels of miR-126 to those of the coding mRNAs in the TCGA cohort (*n* = 719 samples). We performed a Spearman correlation test of miR-126 with all coding genes and applied Bonferroni correction for multiple testing. In Supplementary File 1 we report 661 genes with negative correlation to miR-126 (corrected *p* < 0.05). We then determined the cellular pathways and processes inversely correlated to miR-126 in BC, analyzing the 661 down-regulated genes for enrichment of GO, REACTOME terms and pathways (Supplementary File 1).

**Figure 4:**
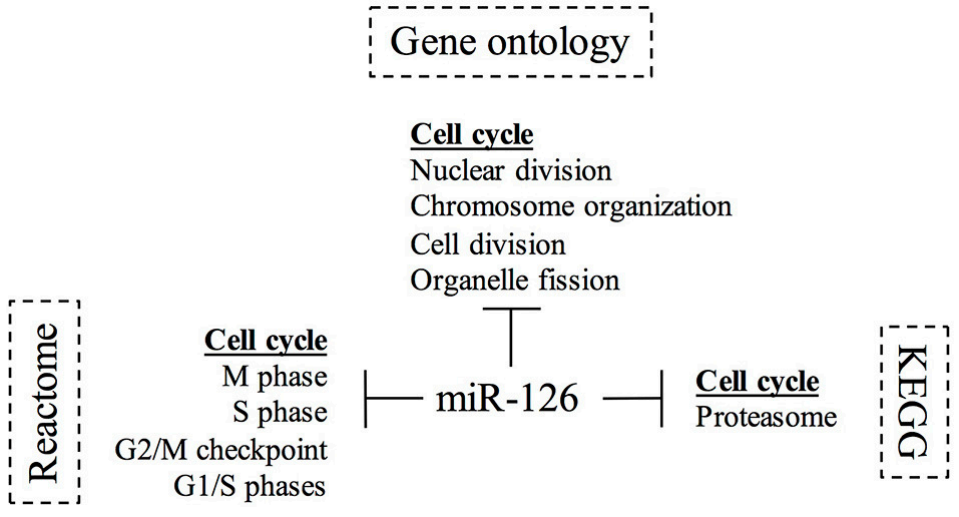
Diagram of Gene ontology and pathways study for miR-126 in breast cancer. The diagram summarizes the involvement of miR-126 in the biological processes and pathways. The KEGG^5^, Reactome^6^, and Gene ontology^7^ databases were interrogated using PantherDB and GSEA.

The REACTOME and GO databases revealed a marked action of miR-126 in down-regulating genes involved in cell cycle, in particular in M phase. The pathway analysis showed a pivotal involvement of miR-126 in DNA replication and glycolysis. We summarized the results from the functional annotation studies in Figure 4, as a diagram reporting the biological process in which miR-126 is involved in breast cancer.

The BC cohort dataset provided a steady state of miR-126 activity. Nevertheless we also wanted to identify the genes possibly directly targeted by miR-126; thus we used a dataset with lentiviral modulation of miR-126 (Lechman et al., 2012). The BC cohort from TCGA provided the bulk of relevant data and the lentiviral experiment (including control, knock-down and over-expression of miR-126) provided the filtering on miR-126 action (Supplementary File 1). In the lentiviral experiment we assigned level 1, −1, and 0, respectively to the over-expressed miR-126, knock-down lentivirus, and control construct. The lentiviral construct is not expressed in BC, thus this analysis is limited, but it shows that in both datasets PIK3R2 (i.e., p85-beta), a regulatory subunit if catalytic p110-alpha subunit (PIK3CA), is negatively associated with miR-126. AKT2, a second reported target of miR126, down-modulated by the lentiviral construct, was not confirmed in the invasive BC cohort. We also investigated the targets for the miRNAs with drugs interaction. Precisely, intersected the predicted target list for each miRNA (from TargetScan v7.1) with that of the cancer genes in Cancer Gene Census (Cosmic v84). This info is reported as Supplementary File 2.

## Discussion

Here we present data supporting the innovative role of treatment with miRNAs in combination with drug in breast cancer. Some studies focused on the effects of miRNA in combination with one or few drugs (Liu et al., 2016; Rui et al., 2017; Strumidło et al., 2017). In our study, we tested 14 drugs, on different subtypes of breast cancer cell lines, in combination with candidate miRNAs. After genome-wide and multi-cohort selection of a pool of candidate miRNAs differentially expressed in BC, we tested their activity on cell line proliferation.

Then we focused on the combination of miRNAs and chemotherapeutic drugs to test for cooperation. As shown in Figure 3, three out of four cell lines revealed some valiant miRNA/drug combinations, namely MCF7, MDA-MB-453, and MDA-MB-468.

In MCF7 cell lines miR-126 confirmed its anti-proliferative role (p = 0.0015), whereas miR-9 and miR-181a reach a *p*-value very close to the significance (*p* = 0.024 and *p* = 0.053, respectively). Other studies supported the anti-tumor role of miR-126 (Zhang et al., 2008; Wang C. Z et al., 2015). In combination with drugs, miR-126 improved the effects of LEE011, GSK1070916, and BYL719. These drugs have targets involved in different pathways: CDK4/6, Aurora b/c, and PI3K, respectively, suggesting multiple ways of action for miR-126. Interestingly, miR-126 is down regulated in breast tumors where the VEGF/PI3K/AKT signaling pathway is activated (Zhu et al., 2011). AZD8055 (*p* = 0.008), AZD5363 (*p* = 0.007), Trametinib, and SCH772984 (*p* = 0.006) drugs, reach a *p*-value very close to the significance. Somehow revealing, still in MCF7, for the ambiguous role of miR-9 in cancer, its positive combination with the ATP-competitive mTORC1/C2 inhibitor drug AZD8055. Although miR-9 alone doesn't show particular effects on MCF7 proliferation, the association with AZD8055 clearly affects the cell growth. The recent founding that miR-9 suppress the expression of the oncogene androgen receptor in MCF7, could suggests a possible explanation of the successful combination with AZD8055 (Moazzeni et al., 2017). Moreover, it has long been known that androgens induces opposite proliferative responses in different cell lines as MCF7 and T47D and this could clear up the different behavior observed in our results (Birrell et al., 1995). By the way, miR-9 is an example of miRNAs for which both tumor suppressor and oncogenic roles have been proposed. In BC for example, the overexpression of miR-9 is found in tumors with aggressive phenotypes and is associated with poor prognosis (Gwak et al., 2014). On the other hand, miR-9, by direct targeting of NOTCH1, can reveal a suppressor-like activity in metastatic breast cancer cells (Mohammadi-Yeganeh et al., 2015) and also epigenetic inactivation of the miR-9 gene has been shown in human BC (Lehmann et al., 2008). Moreover, miR-9 result down regulated in benign breast tumors vs. healthy tissue but overexpressed in malignant breast tumors vs. benign breast tumors suggesting a paradoxical functionality for this miRNA (Hasanzadeh et al., 2016).

The MDA-MB-453 cell line has been chosen as representative of HER2^+^ subclass. It appeared to be the most sensitive cell line to miR/drug combination treatments. The effects of single miRNAs were confirmed for miR-126 and miR-9 (*p* = 0.01 and *p* = 0.003, respectively). miR-126 was active in combination with LEE011, BYL719, AZD8055, AZD5363, Trametinib, and SCH772984. AZD5363, a PKB/AKT isoforms inhibitor, acts like BYL719 and AZD8055 on the PI3K/AKT/mTOR pathway whereas Trametinib and SCH772984, as MEK 1/2 inhibitor and ATP competitive inhibitor of ERK1 and ERK2, respectively, acts on the MEK/ERK pathway. The wide involvement of miR-126 in viability of MDA-MB-453 is entirely coherent with its *in vivo* expression, inversely correlated with HER2 overexpression (Mattie et al., 2006).

miR-326 seemed to have an anti-proliferative effect also as single-agent, but the *p*-value remained only very close to the significance (*p* = 0.058). There are six combinations between miR-326 and drugs with significant interactions on BC viability: LEE011, BYL719, AZD5363, SCH772984, all already seen for miR-126, Gefitinib and Erlotinib, two epidermal growth factor receptor (EGFR) tyrosine kinases inhibitors. Curiously was the behavior of these last two drugs, that alone seemed to have a “proliferative action,” in particular Erlotinib, but they revert their trend in combination with miR-326. Considering HER3 can act as signaling platform when it forms heterodimers with a signaling-competent HER family member (as HER2), this finding can be explained by the observed role of miR-326 in reducing HER3 mRNA and protein levels (Bischoff et al., 2015).

Moreover, what emerged from these combination data is that miR-326, albeit by itself had moderate anti-proliferative effects, in combination treatments can significantly potentiate the activity of BC drugs. Although our data confirmed those of Liang et al. about the involvement of miR-326 in chemotherapy resistance through a negative correlation with multidrug resistance-associated protein (MRP-1), more focused investigation are needed (Liang et al., 2010).

MDA-MB-468 represents in this study the triple negative subtype of BC (TNBC), known to have generally the worst outcome. As TNBC lacks molecular targets, chemotherapy is the only currently available systemic treatment for TNBC, and prognosis remains poor (Foulkes et al., 2010). The alamrBlue assays confirmed the anti-proliferative effects of miR-181a on MDA-MB-468, we observed using MTS (*p* = 0.00036). These are very interesting results indicating a novel, albeit still ambiguous and undefined role, for miR181a in BC. Indeed, for example it has been demonstrated how an up-regulation of miR-181a expression due to TGF-β promotes breast cancer metastasis (Taylor et al., 2013). Moreover, increased expression of miR-181a is correlated with BC aggressiveness and leads to persistence of unrepaired DNA lesions (Bisso et al., 2013). On contrary, Berber et al. observed a down-expression of miR-181a in TNBC in comparison with benign breast tumor (Berber et al., 2014).

Our results showed that miR-181a amplify the effects of GSK1070916, XL765, Doxorubicin, and AMG511. As shown in Table 1 these drugs have completely different targets from each other: Aurora b/c, mTORC1/2, TOPOiso II and PI3K/mTOR, respectively. In accord with these results, Zhu et al. revealed miR-181a as enhancer of doxorubicin-induced apoptosis via targeting Bcl-2 (Zhu et al., 2013). Furthermore, our finding could be supported by a study that reveal how miR-181a can enhances drug sensitivity in BC by targeting breast cancer resistance protein (BCRP/ABCG2) (Jiao et al., 2013).

The two miR-126-LEE011 and miR-126-BYL719 combinations used on MCF7 and MDA-MB-453, showed remarkable effects in both cell lines, but not in T47D and MDA-MB-468 cell lines. This finding suggests that to obtain an efficient combination with the drugs, the activity of the miRNA by itself might be a pre-requirement.

Lastly, our whole transcriptome studies, coupled with gene ontology and pathway analysis suggest an interesting scenario for miR-126 activity in breast cancer. miR-126 appears to be involved in cell cycle regulation, in particular on M phase. Focusing on this role for miR-126, we noted that the drugs LEE011 and BYL719 are also involved in cell cycle inhibition. LEE011 acts directly on CDK4/6, cyclin D1-dependent kinases, which phosphorylate, among other targets, the retinoblastoma protein (Rb) and the related protein p107 and p130, tumor suppressor proteins that allow the cell cycle to proceed from G1 to S phase (Nielsen et al., 1997; Malumbres and Barbacid, 2005). BYL719 inhibits the activity of PI3-kinase alpha (Volinia et al., 1994) a key effector of growth factors' activity, frequently mutated in BC, that leads to the phosphorylation of cyclin D (Diehl et al., 1998). It has been reported that miR-126 is directly involved in PIK3CA pathway, possibly via down-regulation of PIK3R2, one of the p85 regulatory subunits. Lechman et al. showed that miR-126 targets the PI3K/AKT/MTOR signaling pathway, preserving leukemia stem cell quiescence and promoting chemotherapy resistance (Chen et al., 2016; Gao et al., 2016; Lechman et al., 2016; Yang et al., 2017). On the other hand, it has been recently shown a cross talk between CDK4/6 and PI3K inhibitors (Vora et al., 2014; Teo et al., 2017). Further Marcucci and co-workers showed that in chronic myelogenous leukemia miR-126 works in synergism with tyrosine kinase inhibitors (Zhang et al., 2018).

In conclusion, our results confirm that a combination treatment involving miRNAs and drugs can enhance drug activity for specific and important cancer targets. Our experiments further unravel the effects of non-coding RNAs on the potency of BC drugs and open up questions about the cellular mechanisms of interaction that might themselves address novel drug targets.

## Author contributions

FB, SV, MP study concept and design; All authors acquisition, analysis, or interpretation of data; FB, SV, MG drafting of the manuscript; FC, LM, CA, MP, CC critical revision of the manuscript for important intellectual content; FB, LM, CZ, SV, MG statistical analysis; SV, CC obtained funding.

### Conflict of interest statement

The authors declare that the research was conducted in the absence of any commercial or financial relationships that could be construed as a potential conflict of interest.

## Footnotes

^1^www.wcrf.org.

^2^ClinicalTrials.gov: www.clinicaltrials.gov. (Accessed April 10, 2017).

^3^EUClinicalTrialRegister: www.clinicaltrialsregister.eu (Accessed April 10, 2017).

^4^Cancer Gene Census. Available online at: http://cancer.sanger.ac.uk/census

^5^http://www.genome.jp/kegg/pathway.html (Accessed *February* 22, 2017).

^6^http://reactome.org/PathwayBrowser (Accessed *February* 22, 2017).

^7^amiGO2: http://amigo.geneontology.org/amigo (Accessed *February* 22, 2017).

## Abbreviations

ABCG2: G2 member of ATP-binding cassette transporter superfamily
AKT: protein kinase B
ATCC: American type culture collection
ATP: adenosine triphosphate
BC: breast cancer
BCRP: breast cancer resistance protein
BP: biological process
CDK 4/6: cyclin-dependent kinase 4/6
DCIS: ductal *in situ* carcinoma
DMEM: Dulbecco's modified Eagle's medium
EGFR: epidermal growth factor receptor
ER: estrogen receptor
ERK 1/2: extracellular signal-regulated kinase1/2
FBS: fetal bovine serum
HER: human epidermal growth factor receptor
IC: inhibition concentration
IDC: invasive ductal carcinoma
KEGG: Kyoto encyclopedia of genes and genomes
MAD: median absolute deviation
MEK 1/2: MAPK/ERK 1/2
miR and miRNA: microRNA
mTOR: mammalian target of rapamicyn
mTORC 1/2: mammalian target of rapamycin complex 1/2
MTS: 3-(4,5-dimethylthiazol-2-yl)-5-(3-carboxymethoxyphenyl)-2-(4-sulfophenyl)-2H-tetrazolium
OD: optical absorbance
PBS: phosphate buffered saline
PI3KCA: phosphoinositide-3-kinase catalytic alpha polypeptide
PKB: protein kinase B
PMS: phenazine methosulfate
PR: progesterone receptor
PS: Penicillin Streptomycin
TGF-beta: transforming growth factor β
TNBC: triple negative breast cancer
TOPOiso II: topoisomerase II.
