# Supplementary material for: Screen for MicroRNA and Drug Interactions in Breast Cancer Cell Lines Points to miR-126 as a Modulator of CDK4/6 and PIK3CA Inhibitors
Source: Front Genet. 2018 May 18;9:174. doi: 10.3389/fgene.2018.00174 (PMC5968201; doi:10.3389/fgene.2018.00174)
Supplement: Supplementary file 1 [file Table_1.docx]

Title of data: miRNA selection


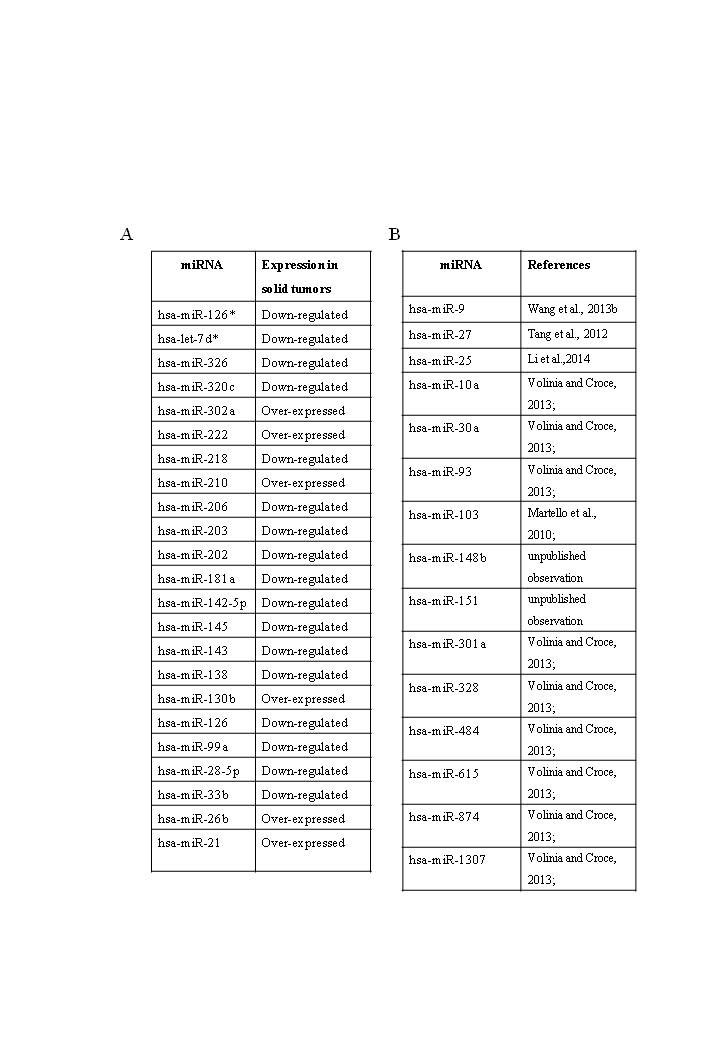


**Supplementary Table 1:** A) The list of miRNAs with differential expression in solid tumors respect to normal tissues. B) The miRNAs associated with prognosis in breast cancer (METABRIC and TCGA cohorts)
