# Supplementary material for: Screen for MicroRNA and Drug Interactions in Breast Cancer Cell Lines Points to miR-126 as a Modulator of CDK4/6 and PIK3CA Inhibitors
Source: Front Genet. 2018 May 18;9:174. doi: 10.3389/fgene.2018.00174 (PMC5968201; doi:10.3389/fgene.2018.00174)
Supplement: Supplementary file 2 [file Table_2.DOCX]

Title of data: Drugs’ concentrations used for each cell line in the miRNA assays

**Supplementary Table 2:** The table shows the drug concentrations used for each cell line. Where no specified, µM is the units of measurement.
