# Supplementary material for: Screen for MicroRNA and Drug Interactions in Breast Cancer Cell Lines Points to miR-126 as a Modulator of CDK4/6 and PIK3CA Inhibitors
Source: Front Genet. 2018 May 18;9:174. doi: 10.3389/fgene.2018.00174 (PMC5968201; doi:10.3389/fgene.2018.00174)
Supplement: Supplementary file 3 [file Table_3.PDF]

Cell line	***Subtype***	***PIK3CA***	***CDKN2A***	***PTEN***	***TP53***	***RB1***	***CDH1***	***BRAF***	***KRAS***
MDA-MB 468	TNBC	wt	wt	**mut**	**mut**	**mut**	wt	wt	wt
MDA-MB-231	TNBC	wt	**mut**	wt	**mut**	wt	wt	**mut**	**mut**
MDA-MB-361	HER+	**mut**	**mut**	wt	**mut**	wt	wt	wt	wt
ZR75.1	Luminal	wt	**mut**	**mut**	wt	wt	wt	wt	wt
MCF7	ER+ PR+	**mut**	**mut**	wt	wt	wt	wt	wt	wt
BT474	Luminal B	**mut**	wt	wt	wt	wt	wt	wt	wt
MDA-MB-453	Luminal B	**mut**	wt	**mut**	wt	wt	**mut**	wt	wt
T47D	Luminal A	**mut**	wt	wt	**mut**	wt	wt	wt	wt
SKBr3	HER+	wt	na	wt	**mut**	wt	**mut (del)**	na	wt
184A1	immortalized normal breast	wt	**mut**	wt	wt	wt	wt	wt	wt
MCF-10A	immortalized normal breast	wt	**mut**	wt	wt	na	na	na	na
HBL-100	immortalized breast myoepithelial	wt	wt	wt	wt	na	na	wt	wt

Supplementary Table 3. The table reports the notable breast cancer genes, as listed in Cancer Gene Census (https://cancer.sanger.ac.uk/census), with somatic mutations in the breast cancer cell lines used in this study
