# Supplementary material for: Screen for MicroRNA and Drug Interactions in Breast Cancer Cell Lines Points to miR-126 as a Modulator of CDK4/6 and PIK3CA Inhibitors
Source: Front Genet. 2018 May 18;9:174. doi: 10.3389/fgene.2018.00174 (PMC5968201; doi:10.3389/fgene.2018.00174)
Supplement: Supplementary file 6 [file Image_1.PDF]

Title of data: Combinations of miR-126 with LEE011 or BYL719 on T47D and MDA-MB-468 cell lines

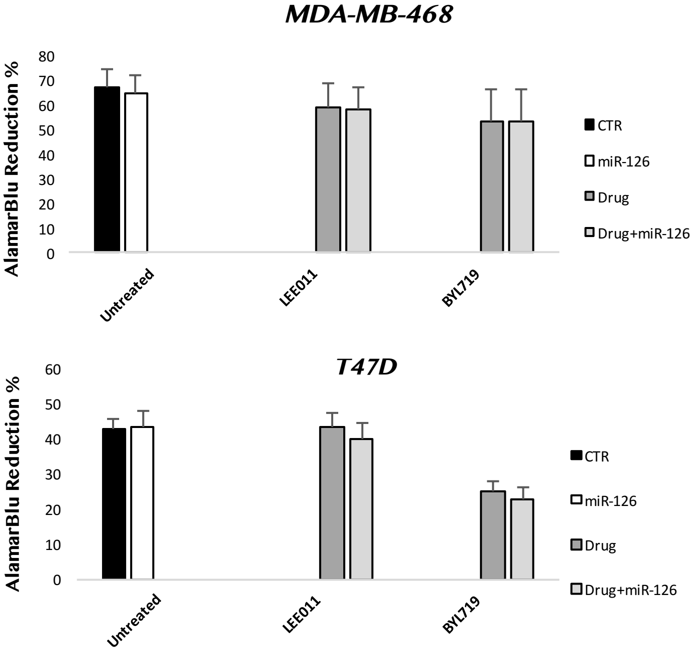

Supplementary Figure 1: miR-126-LEE011 and miR126-BYL719 effects on cell viability for T47D and MDA-MB-468.
