# Supplementary material for: Screen for MicroRNA and Drug Interactions in Breast Cancer Cell Lines Points to miR-126 as a Modulator of CDK4/6 and PIK3CA Inhibitors
Source: Front Genet. 2018 May 18;9:174. doi: 10.3389/fgene.2018.00174 (PMC5968201; doi:10.3389/fgene.2018.00174)
Supplement: Supplementary file 7 [file Image_2.PDF]

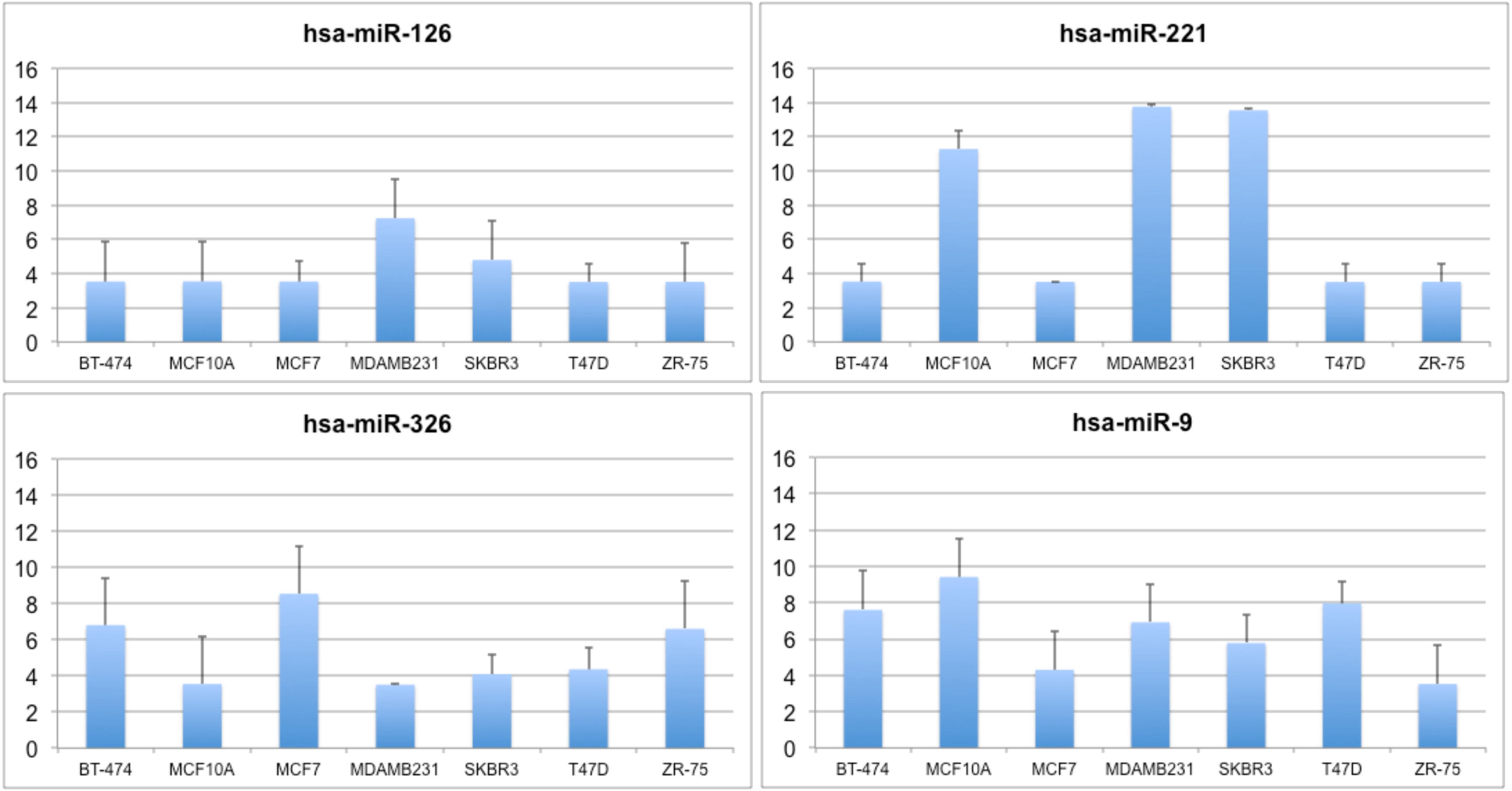

Supplementary Figure 2. The expression of miR-126, miR-326, miR-9 and miR-221 in breast cancer cell lines. The log2 levels of microRNA expression are shown on the Y axis.
